# Complementary processing of haptic information by slowly and rapidly adapting neurons in the trigeminothalamic pathway. Electrophysiology, mathematical modeling and simulations of vibrissae-related neurons

**DOI:** 10.3389/fncel.2013.00079

**Published:** 2013-06-04

**Authors:** Abel Sanchez-Jimenez, Carlos Torets, Fivos Panetsos

**Affiliations:** ^1^Department of Applied Mathematics (Biomathematics), Faculty of Biology, Complutense University of MadridMadrid, Spain; ^2^Neurocomputing and Neurorobotics Research Group, Instituto de Investigación Sanitaria del Hospital Clínico San Carlos, Universidad Complutense de MadridMadrid, Spain; ^3^Department of Applied Mathematics (Biomathematics), School of Optics, Complutense University of MadridMadrid, Spain

**Keywords:** vibrissa, somatosensory system, sensory trigeminal complex, cortex

## Abstract

Tonic (slowly adapting) and phasic (rapidly adapting) primary afferents convey complementary aspects of haptic information to the central nervous system: object location and texture the former, shape the latter. Tonic and phasic neural responses are also recorded in all relay stations of the somatosensory pathway, yet it is unknown their role in both, information processing and information transmission to the cortex: we don't know if tonic and phasic neurons process complementary aspects of haptic information and/or if these two types constitute two separate channels that convey complementary aspects of tactile information to the cortex. Here we propose to elucidate these two questions in the fast trigeminal pathway of the rat (PrV-VPM: principal trigeminal nucleus-ventroposteromedial thalamic nucleus). We analyze early and global behavior, latencies and stability of the responses of individual cells in PrV and medial lemniscus under 1–40 Hz stimulation of the whiskers in control and decorticated animals and we use stochastic spiking models and extensive simulations. Our results strongly suggest that in the first relay station of the somatosensory system (PrV): (1) tonic and phasic neurons process complementary aspects of whisker-related tactile information (2) tonic and phasic responses are not originated from two different types of neurons (3) the two responses are generated by the differential action of the somatosensory cortex on a unique type of PrV cell (4) tonic and phasic neurons do not belong to two different channels for the transmission of tactile information to the thalamus (5) trigeminothalamic transmission is exclusively performed by tonically firing neurons and (6) all aspects of haptic information are coded into low-pass, band-pass, and high-pass filtering profiles of tonically firing neurons. Our results are important for both, basic research on neural circuits and information processing, and development of sensory neuroprostheses.

## Introduction

Tactile perception during haptic exploration of an object along with sensory feedback for motor control during object manipulation require precise information about various complex spatiotemporal parameters such as shape, roughness, position, direction, acceleration, contact forces, etc. By sweeping their fingers at 4–12 Hz across the objects, primates code the above spatiotemporal parameters in the deflections and vibrations of the skin (Johnson, 2001). A similar process is observed in rodents. In this case the principal tactile apparatus are the whiskers and haptic information is encoded in their deflections and vibrations when are swept at the same 4–12 Hz frequency across the objects (Carvell and Simons, 1990; Salinas et al., 2000; Sachdev et al., 2001; Bermejo et al., 2002). In primates low-frequency skin motion and object location are transmitted by the fibers of the rapidly adapting ganglion cells (phasic-Ph) while high-frequency skin motion, form and texture are transmitted by the slowly adapting ones (tonic-T cells) (Blake et al., 1997; Dodson et al., 1998; LaMotte et al., 1998; Wheat and Goodwin, 2001; Goodwin and Wheat, 2004). The same separate channels are present in rodents' tactile primary afferent fibers (Zucker and Welker, 1969; Duc et al., 1994; Baumann et al., 1996; Shoykhet et al., 2000; Leiser and Moxon, 2006).

A very important and currently unanswered question regards the existence of two separate information channels alongside the somatosensory pathway and to what extent Ph and T neural populations process and transmit complementary information to the cortex. The use of separate Ph and T neurons for the transmission of haptic information submodalities in the periphery and the massive presence of Ph and T neurons in all relay stations of the central somatosensory pathway (brainstem, thalamus, cortex) suggest the existence of a more general information processing and coding strategy based on the existence of a two-channel information pathway also in the central nervous system. However, Jones et al. (2004) did not find differences in the mean spiking rates and timing of trigeminal ganglion Ph and T cells and almost all identified thalamic projection neurons recorded by Minnery and Simons (2003) were T.

In the present work we proposed to answer this question in the case of the brainstem principal nucleus (PrV) of the rodent whisker-trigeminal system. Since both, slowly and rapidly adapting primary afferents relay on PrV neurons, is essential to reveal coding strategies used by these cells because they will condition processing and coding mechanisms in the subsequent stations of the sensory pathway.

By combining single-unit electrophysiological recordings, mathematical modeling and numerical simulations we (1) elucidated the role of PrV Ph and T neurons in the processing of frequency-dependent tactile stimulation, (2) rejected the two channels hypothesis for the transmission of sensory information to the thalamus, (3) obtained evidence that both, Ph and T responses, are originated from a unique type of PrV cell, (4) determined that these PrV neurons do not respond phasically or tonically due to intrinsic PrV nuclear dynamics but to a differential excitatory and inhibitory modulation exerted by the sensorimotor cortex.

## Materials and methods

All experiments were carried out according to EU Directives (86/609/EC) and national legislation (R.D. 1201/2005, Ley 32/2007) with regards to this matter, trying to reduce the number of sacrificed animals and to avoid suffering. Data were obtained from 52 urethane-anesthetized (1.5 g/kg i.p.) adult albino Wistar rats of either sex weighing 280–320 g. Experimental procedures are detailed in Sanchez-Jimenez et al. (2009). A brief description of the preparation, stimulation, recording and data analysis is detailed below.

### Animal preparation, vibrissae stimulation and recordings

Animals were placed in a stereotaxic device (Narishige Co., LTD., Japan, model SN-3N) and the scalp was removed. For PrV recordings the bone was opened 2.5–3.2 mm lateral to the midline and 8.8–9.2 mm posterior to the Bregma. For lemniscal recordings a hole was made 1.80–1.90 mm lateral to the midline (contralateral to the stimulated vibrissae) and 5.00–5.40 mm posterior to the Bregma. For EEG, the hole was made in the frontal part of the skull. To remove cortical influence the contralateral sensorimotor cortex was aspired using a Pasteur micropipette connected to a vacuum pump (Vacumsol AS-60, ALSA Apparecchi Medicali S.R.L., Italy). After the removal of the cortical tissue the hole was covered with Vaseline oil and animals were left for 3–4 h before starting the recordings. At the end of each experiment the position of the recording site was determined by inducing electrolytic lesions passing 3–5 mA, 5 s-long currents through the tip of the electrode. Rats were then sacrificed by an overdose of sodium pentobarbital (50 mg/kg) and brains were removed, immerged in cryogenic solution and stored at −20°C for a posteriori histological processing. Some animals were transcardially perfused with saline followed by formalin (4% in saline). Brains were stored in 20% sucrose saline and cut on a freezing microtome into 50 μm coronal sections that were then stained by Nissl or cytochrome oxidase (CyO) to locate the recording sites.

Distal portions (i.e., free ends) of vibrissae were stimulated using air-jets generated by a pneumatic pressure pump (10 psi, Picospritzer III, Parker, Texas, USA) and delivered in a rostro-caudal direction via a 0.5 mm diameter silicon tube positioned at a distance of 10–12 mm from the vibrissa. Once the principal vibrissa was identified, spontaneous activity was recorded for 180 s and then the 1–40 Hz stimulation protocol was followed: 5 s long trains of 14 ms-long air-puffs at 1, 2, 3, 5, 8, 10, 12, 15, 20, 25, 30, 35, and 40 Hz were presented 10 times in a random order with a 3 s interval between trains (Garabedian et al., 2003; Sanchez-Jimenez et al., 2009). Experiments terminated with a final sequence of 50 pulses of 100 ms at 1 Hz. Lemniscal recordings were obtained from a reduced version of this protocol: 2 s long trains of 14 ms-long air-puffs at 3, 5, 8, 10, 12, 15, 20, 30, and 40 Hz.

PrV extracellular recordings were obtained using 0.8–2.0 MΩ tungsten microelectrodes (World Precision Instruments, Inc.) placed in the zone of the barrelets ipsilaterally to the stimulated whisker. The location of the electrodes was estimated by the stereotaxic coordinates and also inferred from the stereotyped somatotopy of each nucleus. Lemniscal recordings were obtained using 5.0 MΩ tungsten microelectrodes. Once a neuron was isolated, its receptive field was manually determined and the whisker that elicited the maximum activation was labeled as the principal whisker. EEG recordings were obtained from an isolated Cr-Ni wire (125 μm diameter) inserted 1.0 mm deep into the frontal cortex and fixed with dental cement. Recorded signals were amplified, filtered online (0.3–3.0 KHz, DAM80 bio amplifier, World Precision Instruments, Inc.), digitalized (300 Hz EEG recordings, 20 KHz extracellular recordings. 1401 mkII Digidata, Cambridge Electronic Design) and stored in hard discs for off-line analysis.

### Data analysis and statistics

Neural responses were analyzed by means of functions related to coding of different object characteristics such as location (early behavior), movement (temporal consistency and latency), form and texture (global behavior), etc., all of them at increasing stimulation frequencies (Garabedian et al., 2003; Sanchez-Jimenez et al., 2009). Taking into account whisker dynamics we divided the 1–40 Hz range in three classes: whisking (*W*_IN_, 4–14 Hz), below whisking (*W*_BE_, <4 Hz) and above whisking (*W*_AB_, >14 Hz).

**Figure 1:**
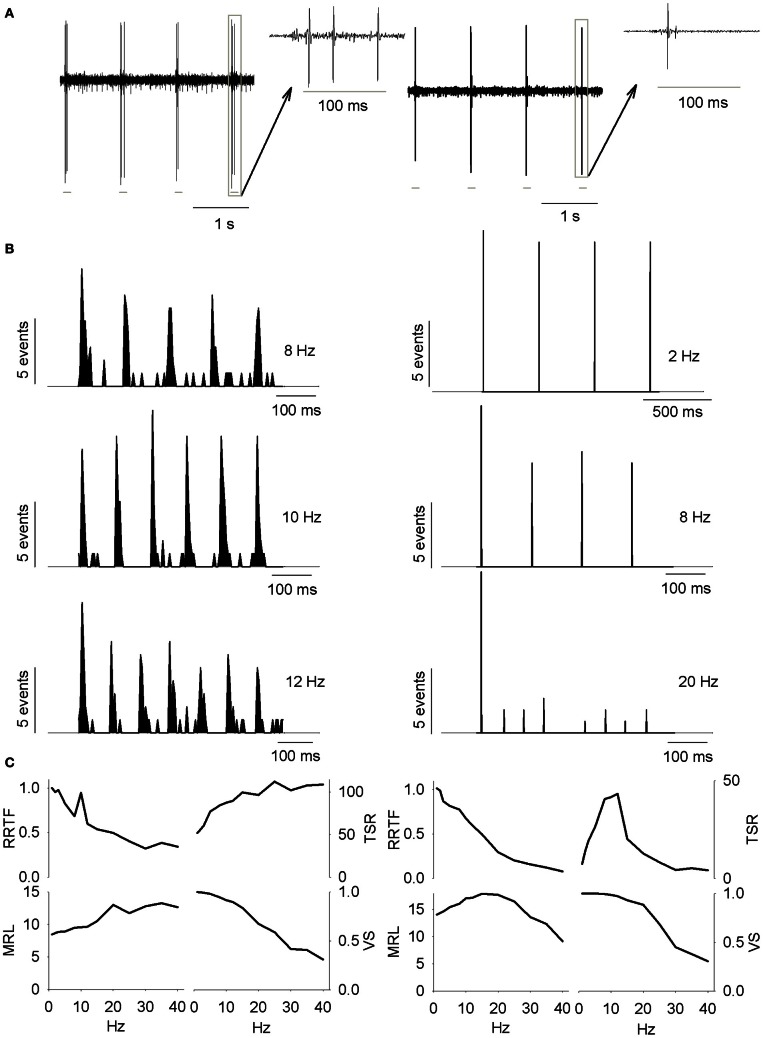
**(A)** PrV tonic (T-left) and phasic (Ph-right) responses to four air-jet stimuli. Insets show the responses to an individual stimulus. Gray lines below the recordings indicate the duration of the stimuli. **(B)** Peristimuli histograms of the neural responses illustrated in **(A)**. Three different stimulation frequencies are shown. Tonic responses (left) display a clear potentiation at 10 Hz while phasic ones reduce the number of spikes with the increase of the stimulation frequency. **(C)** Response rate transfer function, RRTF; total spike response, TSR; mean response latency, MRL; and vector strength, VS; profiles of the above neural responses. Tonic response is band-pass, BP; in early response, RRTF; high-pass, HP; in global response, TSR; and low-pass, LP; in mean response latency and temporal consistency (MRL-VS). Phasic response is LP in RRTF and VS and BP in TSR and MRL. Attention: tonic MRL is LP because latencies increase with stimulation frequency! X-axes represent stimulation frequency.

Early behavior was characterized by the Repetition Rate Transfer Function (RRTF). At each stimulation frequency *f*, *f* ∈ (1, 40 Hz) we deliver *n*(*f*) stimuli during the 5 seconds-long stimulation trains: *n*(1) = 6, *n*(2) = 11, etc.,

$$\text{RRTF }(f)=\frac{\sum_{j=2}^{n(f)}\frac{sp(j)}{n(f)-1}}{sp(1)}$$

where *sp*(*j*), *j* = 1, *n* represent the number of spikes elicited in the first 15 ms after the onset of the *j*-th stimulus of the train. RRTF >1 indicates potentiation while RRTF <1 indicates adaptation of the response. For global behavior we considered the total number of spikes evoked by the entire 5-s train (Total Spike Rate-TSR). To compare recordings we normalized TSR values along the 1–40 Hz range by the value of the frequency with the highest TSR (TSRn). Mean response latency (MRL) was defined as the post-stimulus time at which the response amplitude reached 50% of its peak value, evaluated via the average cycle histograms of all but the first stimuli of a train. To properly compare neural responses MRL were normalized with respect to the maximum value among stimulation frequencies (MRLn). Finally, temporal consistency was evaluated by the stability of the phase-locking of the responses to the external stimuli by means of the vector strength function (VS):

$$VS=\frac{\sqrt{{\left(\sum_{i=1}^{n}\cos\left(\theta_{i}\right)\right)}^{2}+{\left(\sum_{i=1}^{n}\sin\left(\theta_{i}\right)\right)}^{2}}}{n}$$

considering each *i*-th spike as a vector of unitary length and argument $\theta_{i}=2\pi (\frac{t_{i}}{T}),\;0\leq \theta \leq 2\pi$, where “*n*” is the total number of spikes evoked during the stimulus train, *T* is the period of the stimulus frequency and *t*_*i*_ is the time interval between the most recent vibrissa deflection and the *i*-th spike (Goldberg and Brown, 1969). VS takes values between 0 and 1, from random spiking to perfect phase-locking. HF and LP neurons displayed opposite exponential behaviors (see Figure 1C); neural cells were characterized as BP if values were ≥20% of their neighborhoods (e.g., Figure 1C) at some specific frequency; NF when function values were similar at any frequency (differences ≤10%).

Data analysis was performed by custom software written in Spike2 software (Cambridge Electronic Design) and Matlab© script language. Spikes were threshold-isolated offline and converted into discrete processes. A first analysis was performed by means of the at-rest spiking rate and peristimulus histograms (PSTHs) with a time resolution of 1 ms.

Data are shown as mean ± standard error of the mean (SEM). Statistical comparisons were performed by means of Mann-Whitney (Wilcoxon) *W*-test. To correct for multiple comparisons the Benjamini-Hochberg-Yekutieli false discovery rate procedure was used. To analyze differences in the distribution of categorical data (proportions) independence χ^2^ tests were used, which were substituted by exact Fischer's test for 2 × 2 tables when required by the sample size. For cluster analysis *k*-means algorithm (distance measure squared euclidean) was used. The correct number of clusters within each dataset (*k*) was determined by means of silhouette plots and averages. Statistical tests were performed using Statgraphics Centurion XVI and Matlab© softwares. Significance level (α) was set to 0.05 in all cases.

### Simulation of spike trains

For the mathematical study we developed a stochastic spiking model representing PrV tonic and phasic neurons as inhomogeneous Poisson processes. Each stimulus generates an arbitrary sequence of “*n*” spike times within stimulation period *T*_*f*_, the probability density for these “*n*” ordered spike times at each stimulation frequency “*f*” being

$$P_{f}[t_{1},\;t_{2},\;\ldots ,\;t_{n}]=\exp(-\int_{0}^{T_{f}}r(t,\;f)dt)\prod_{i=1}^{n}r(t_{i},\;f)$$

where *r*(*t*, *f*) represents the time-varying firing rate which depends on stimulation frequency.

For each recorded PrV neuron we created a simulation based on its own *r*_0_(*t*), RRTF(*f*), TSR(*f*), MRL(*f*), and VS(*f*) values. This way we subdivide time into 1-ms bins and for each bin we generate a random number “*x*” uniformly distributed between 0 and 1. If the estimated firing rate is greater than this random number, then a spike is fired. Otherwise no spike is generated. Firing rate for the first stimulus of each 5-s series [*r*_0_(*t*)] does not depend on stimulation frequency. Firing rate for subsequent stimuli at each stimulation frequency *r*(*t*, *f*) was calculated as a function of *r*_0_(*t*), RRTF, TSR, VS, and MRL. First we set initial latency (MRL_0_) as the MRL obtained from the final sequence of 50 stimuli at 1 Hz. Response began at a time *t*_1_(f) = MRL(f)—MRL_0_ so *r*(*t*, *f*) at earlier times (between 0 and *t*_l_–1 ms) was zero and between *t*_1_ and 15 ms depended only on RRTF(*f*)

$$r(t,\;f)=r_{0}(t)\times \text{RRTF}(\text{f}),{\text{ t}}_{1}\leq t\leq 15\text{ ms}$$

at times after 15 ms *r*(*t*, *f*) depends on both TSR(*f*) and RRTF(*f*)

r(t, f)=r0(t)×TSR(f)−TSR(1)nstimnstim−1−TSR(1)nstim×RRTF(f),   t>15 ms

Once we have simulated spike trains, we generated a random number uniformly distributed between 0 and 1 for each stimuli. If this number for a certain stimulus was greater than the value of VS at the corresponding frequency spikes evoked by the stimulus were displaced from their original bin following a standard normal distribution.

To simulate cortex removal and excitation of decorticated recordings we compute the difference between mean *r*_0_(*t*) of the whole set of neurons from intact animals and mean *r*_0_(*t*) of the whole set of neurons from decorticated animals. This mean difference was then added or subtracted from the real *r*_0_(*t*) of each recording.

Simulations were performed by means of custom software written in Matlab© script language.

## Results

### Nucleus principalis. complementary information processing by tonic and phasic neurons

First of all, we investigated to what extent T and Ph cells could be considered as two different information processing structures into PrV. For this reason we recorded 144 individual neurons (105 T and 39 Ph, 73 and 27%, Figure 1A) from the barrelets region of 32 intact animals and we studied their early and global behavior, response latencies and temporal consistency to 1–40 Hz tactile stimuli. Neurons behaved in a frequency-dependent manner for all response variables performing low-pass (LP), high-pass (HP), or band-pass (BP) potentiation/filtering. A small number of neurons did not perform any type of filtering (NF). Interestingly T and Ph cells always performed in a highly complementary manner. Representative peristimuli histograms under different stimulation frequencies are shown in Figure 1B and filtering profiles in the 1–40 Hz range are shown in Figure 1C.

#### Early behavior

**Figure 2:**
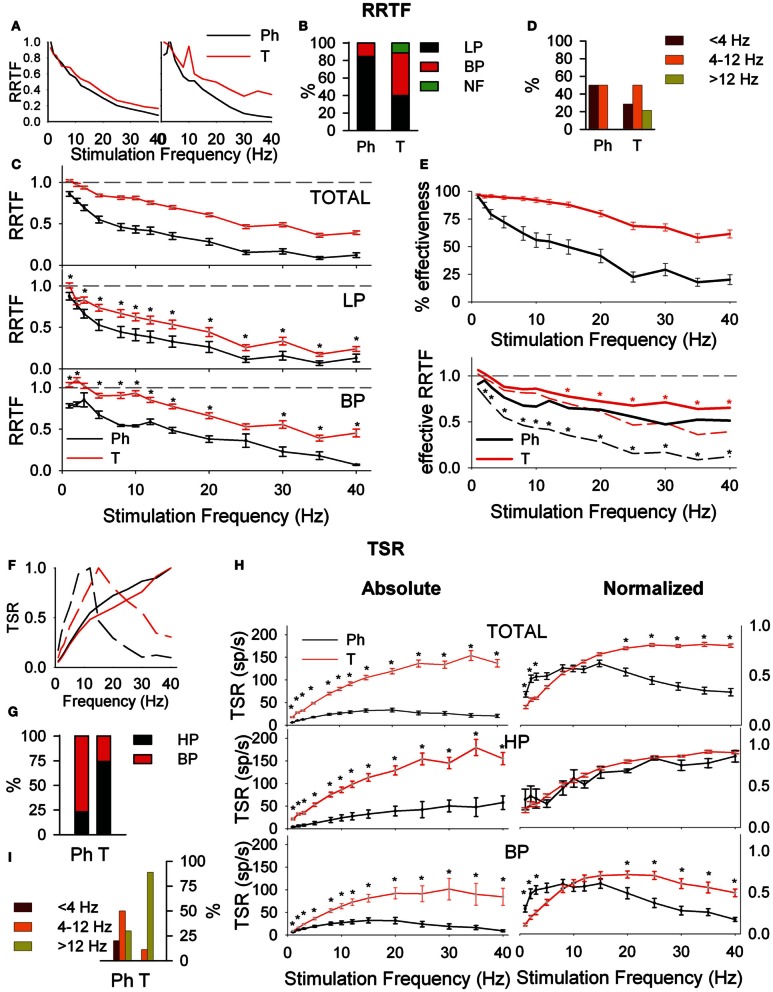
**Early (A–E) and global (F–I) behavior of tonic (T) and phasic (Ph) PrV neurons. (A)** Typical early response functions of low-pass (LP, left) and band-pass (BP, right) cells. **(B)** Percentages of LP and BP populations among Ph and T neurons. **(C)** Mean repetition rate transfer function (RRTF) values for Ph and T responses pooled all together (top) and considering LP and BP separately (middle and bottom). **(D)** BP neurons were characterized by an increase of RRTF at a certain frequency (peak frequency). Bar plot shows the distribution of these peak frequencies within the three frequency ranges [below (<4 Hz), into (4–12 Hz) and above (>12 Hz) whisking] for Ph and T groups separately. **(E)** Effectiveness of the delivered stimuli to Ph and T neurons (top) and RRTF function calculated using only effective stimuli (bottom). **(F)** Typical total spike response functions (TSR) of high-pass (HP, continuous lines) and BP (dashed lines) cells. **(G)** Percentages of HP and BP populations among Ph and T neurons. **(H)** Mean absolute (left) and normalized (right) TSR values for Ph and T responses pooled all together (top) and considering HP and BP separately (middle and bottom). **(I)** Distribution of BP peak frequencies for Ph and T neurons in the three frequency ranges: below (<4 Hz), into (4–12 Hz), and above (>12 Hz) whisking. (^*^) indicate adjusted *p*-values <0.05 (FDR procedure, 13 comparisons). In **(E)** red stars correspond to comparisons of tonic neurons and black stars to phasic.

It is given by the RRTF that indicates if repeated stimulation potentiates or not the neural responses (if the second, third, etc., stimulus provokes a higher number of early spikes compared to the first one). RRTF >1 indicates potentiation while RRTF <1 indicates adaptation of the response. PrV neurons adapt their RRTF values following LP or BP profiles (Figure 2A). Ph neurons are mostly LP (85%) while T neurons are either LP or BP (40–49%, Figure 2B). T neurons show significantly higher RRTF values (lower adaptation) at all stimulation frequencies, both globally and for LP-BP neurons considered separately (see Figure 2C. The two non-significant differences among BP neurons were due to the low number of Ph neurons). Ph and T neurons differ also in the distribution of their BP potentiation frequencies: Ph potentiate in *W*_BE_ and filter in *W*_IN_ (6 frequencies) and T potentiate over the whole 1–40 Hz range (84 frequencies) as shown in Figure 2D.

Adaptation could be either due to a decrease of the number of spikes elicited by the second, third, etc., stimuli or to a decrease of the number of stimuli that elicit neural responses (effective stimuli). To determine the contribution of these two sources we calculated the effectiveness of our neurons, as the number of effective stimuli at each stimulation frequency. Ph effectiveness follow an exponential decrease while T ones are very stable with >90% in *W*_BE_−*W*_IN_ (Figure 2E, top). Effective Ph RRTF is higher than the crude one in the whole stimulation range and T one in *W*_AB_ (Figure 2E, bottom).

#### Global behavior

It is represented by the total number of spikes evoked by the entire 5-s train (Total Spike Rate-TSR). TSR values show HP and BP profiles (Figure 2F). TSR display a high complementarity with Ph neurons being BP and T being HP (77 and 74%, respectively, Figure 2G). Because of their lower response spiking rates, Ph neurons show significantly lower TSR values at all stimulation frequencies, both globally and for separate LP-BP neurons (Figure 2H, left). To overcome this bias we considered normalized TSR values along the 1–40 Hz range by the value of the frequency with the highest TSR (TSRn). Ph and T neurons differ in BP profiles outside *W*_IN_ (Figure 2H, right-bottom) and in the distribution of their BP potentiation frequencies (Figure 2I): Ph potentiate in the whole 1–40 Hz range (30 frequencies) while T potentiate almost exclusively in *W*_AB_ (24 out of 27 frequencies).

#### Response latency (MRL)

**Figure 3:**
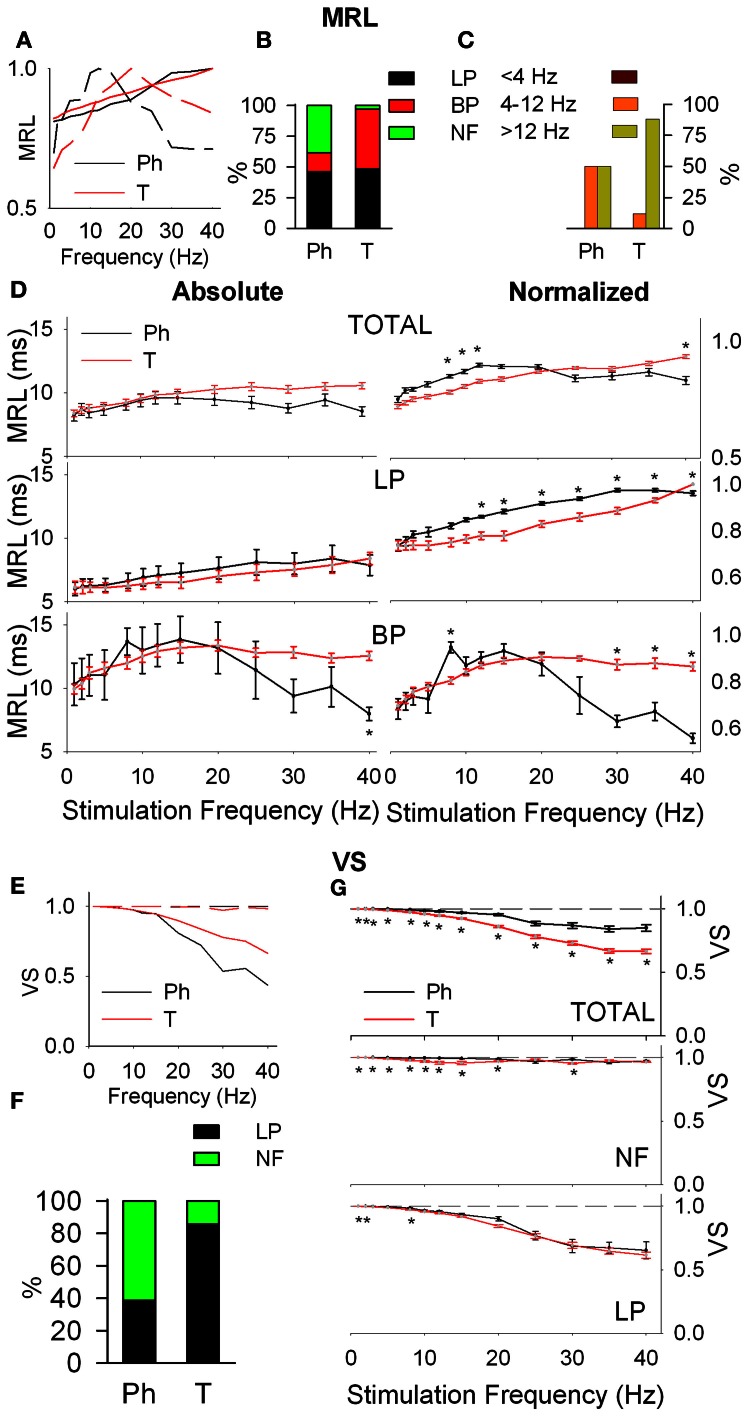
**Response latency (MRL, A–D) and temporal consistency (VS, E–G) of tonic (T) and phasic (Ph) PrV neurons. (A)** Typical response latency functions of low-pass (LP, continuous lines) and band-pass (BP, dashed lines) cells. **(B)** Percentages of LP and BP populations among Ph and T neurons. **(C)** Distribution of BP peak frequencies for Ph and T neurons in the three frequency ranges: below (<4 Hz), into (4–12 Hz), and above (>12 Hz) whisking. **(D)** Mean absolute (left) and normalized (right) MRL values for Ph and T responses pooled all together (top) and considering LP and BP separately (middle and bottom). **(E)** Typical temporal consistence functions of LP (continuous lines) and no-filtering (NF, dashed lines) cells. **(F)** Percentages of LP and NF populations among Ph and T neurons. **(G)** Mean VS values for Ph and T responses pooled all together (top) and considering LP and BP separately (middle and bottom). (^*^) indicate adjusted *p*-values < 0.05 (FDR procedure, 13 comparisons).

Response latency (MRL), defined as the delay between the onset of the stimulus and the appearance of a significant response of the neuron show LP, BP, and NF profiles (Figure 3A). Ph and T neurons are complementary in BP-NF percentages: 15–39% the former vs. 49–12% the later (Figure 3B) and differ in the distribution of their BP potentiation frequencies (Figure 3C): Ph potentiate in *W*_IN_ and *W*_AB_ (3 cases each) while T potentiate in the *W*_AB_ range (88%, 45 out of 51 frequencies). Ph and T neurons show similar filtering profiles both globally and when LP-BP responses are considered separately (Figure 3D, left). This is not true in normalized values (relative changes, normalized with respect to the maximum value among stimulation frequencies, Figure 3D, right) where at high stimulation frequencies Ph neurons increase the MRL of LP responses and decrease that of BP.

#### Temporal consistency

Temporal consistency offered us an estimation of the stability of the neural responses alongside the stimulation trains (VS takes values between 0 and 1, corresponding to random spiking and perfect phase-locking, respectively). VS values show LP and NF profiles (Figure 3E) with a 62% of Ph and an 86% of T cells being LP (Figure 3F). Although temporal consistency is extremely high for all PrV neurons (VS very near 1.0) specially below 15 Hz, Ph responses are significantly more stable in the whole 1–40 range (Figure 3G).

The above results provide experimental evidence that trigeminal Ph and T neurons play a complementary role in the processing of complex tactile stimuli and each of them could participate to a different information processing strategy and even to a different subnetwork. However, the above data do not provide any support that Ph and T neurons also constitute, or even belong, to separate pathways that convey complementary information to the cortex. Neither provide support that the two response profiles are also originated by two different cellular morphologies. The next step was oriented to test the “two pathways” hypothesis and to elucidate the origin of Ph and T responses.

### Information transmission to the thalamus. testing the “two pathways” hypothesis

The two pathways hypothesis considers that there are two different types of neurons, Ph and Th that transmit information to the thalamus. If the “two pathways” hypothesis is true, Ph and T neurons transmit their information concurrently to the thalamus. Taking into account that PrV projects to the thalamus through the medial lemniscus (LEM) we expect LEM fibers to be characterized by similar activity to the above PrV recordings (made in the barrelets region populated by thalamic projecting neurons). To test this hypothesis we recorded the responses of 49 individual LEM fibers from 6 additional animals under 1–40 Hz air-jet stimulation of the whiskers.

**Figure 4:**
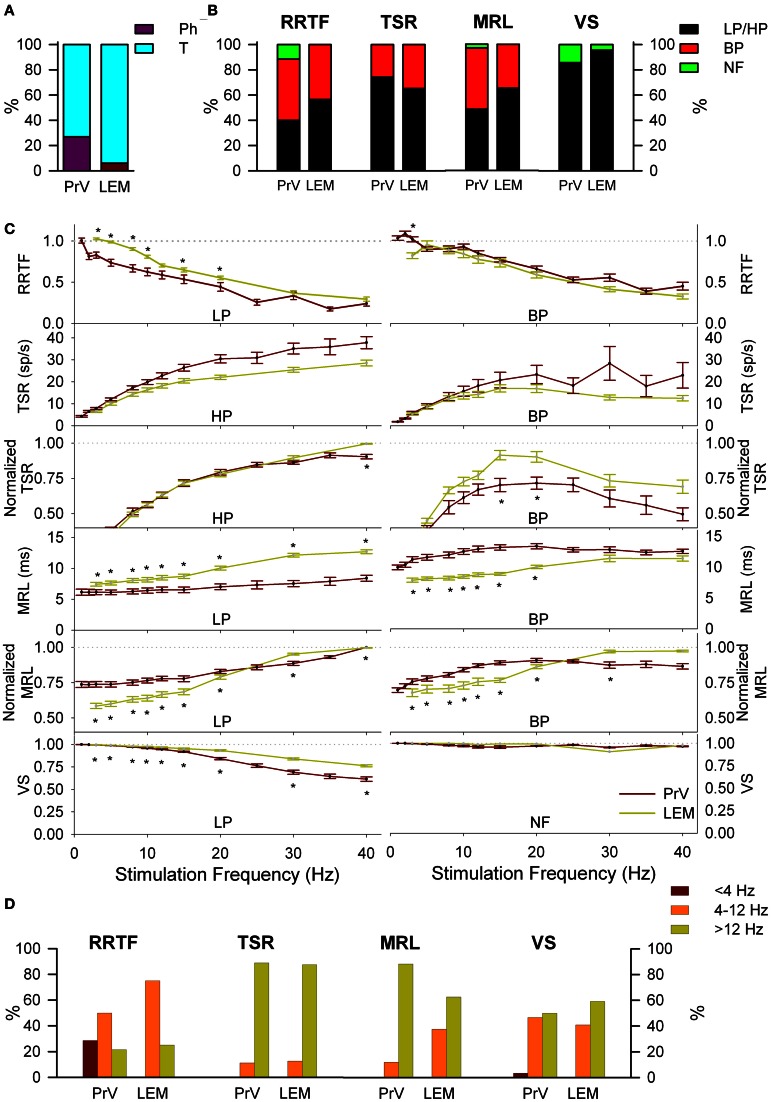
**Comparison between PrV and lemniscal (LEM) recordings. (A)** Percentages of phasic (Ph) and tonic (T) responses in PrV and lemniscal recordings. **(B)** Percentages of the different filtering profiles for repetition rate transfer function (RRTF), total spike response (TSR), mean response latency (MRL), and vector strength (VS). **(C)** Mean RRTF, TSR, MRL, and VS values of low-pass (LP) or high-pass (HP) (left) and band-pass (BP) profiles (right) of PrV and lemniscal recordings. Significant differences suggest that not all T PrV neurons project to the thalamus. **(D)** Distribution of BP frequencies for PrV and lemniscal recordings in the three frequency ranges: below (<4 Hz), into (4–12 Hz) and above (>12 Hz) whisking. Comparisons in **(B)** and **(C)** take into account only tonic responses since lemniscal recordings were almost exclusively tonic as shown in **(A)**. (^*^) indicate adjusted *p*-values < 0.05 (FDR procedure, 13 comparisons).

LEM recordings did not confirm the “two pathways” hypothesis. Firstly, LEM recordings were almost exclusively T (94%) in contrast to the 27% Ph and 73% T of the PrV ones (Figure 4A). Secondly, although lemniscal T responses displayed LP-BP-HP profiles and distributions similar to Tonic PrV ones for all four variables (see Figure 4B) they also showed several significant differences (Figures 4C,D): (1) higher RRTF values of LP responses for <25 Hz stimulation frequencies; (2) larger LP and BP MRL although lemniscal recordings were performed at a longer distance than PrV ones, 1–40 Hz for LP and <25 Hz for BP profiles and (3) higher stability of LP responses.

The above results suggest that information transmission to the thalamus is performed only by a subset of T trigeminal neurons. Taking into account that our recordings were randomly made in the zone of thalamic projecting neurons, recordings from these specific neurons should be included into the totality of recordings. To identify this specific group we performed a *k*-means group separation of the totality of our PrV recordings taking into account the values of the four functions and the filtering profiles. Neural activity of the thalamic projecting neurons had to be statistically similar to that recorded from the medial lemniscus (RRTF-LP, RRTF-BP, TSR-HP, TSR-BP, MRL-LP, MRL-BP, and VS-LP tonic profiles) and different from the remaining PrV cells.

Group separation was carried out in two steps.

**Figure 5:**
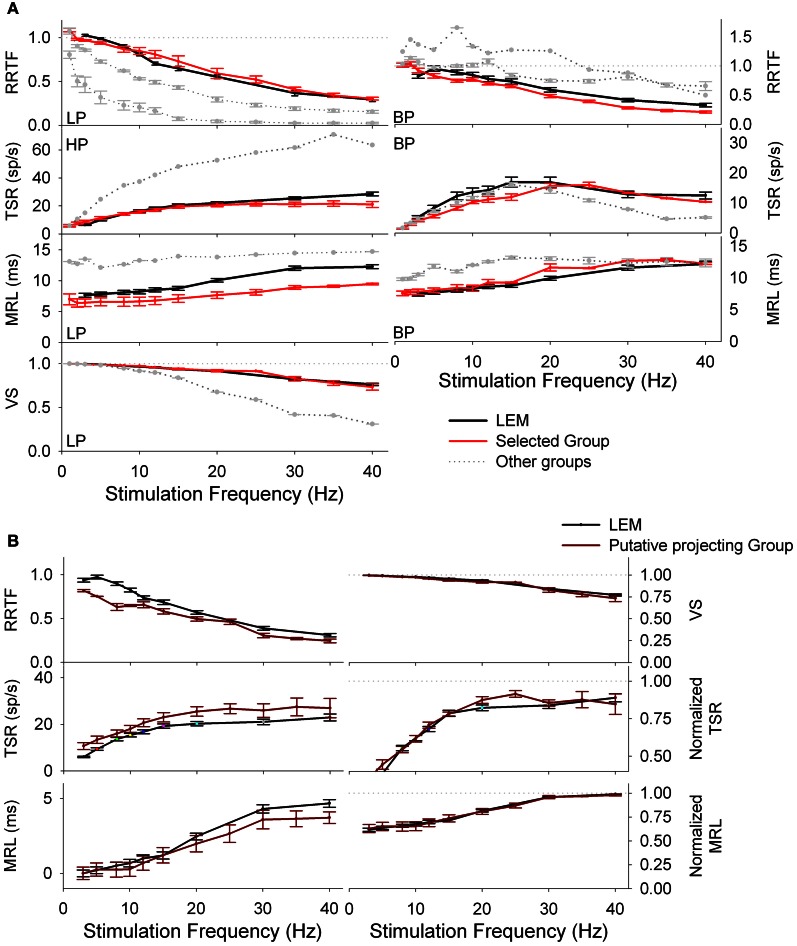
**Group separation of tonic PrV recordings according to the type of the response (LP/HP or BP). (A)** RRTF, TSR, MRL, and VS values of tonic PrV responses are clustered; for each stimulation frequency mean values are calculated and plotted (in gray-black) together with the mean values of the lemniscal recordings (in red). PrV clusters closest to the lemniscal ones (in black) are expected to contain the thalamic projecting neurons. However, not all recordings in black clusters correspond to thalamic projecting neurons since many neurons display values in the black cluster for one variable and values in a gray cluster for another. **(B)** Mean values of PrV neurons whose RRTF, TSR, MRL, and VS values belong (all of them) to the black clusters plotted in **(A)**. The values of these neurons are very similar to those of the lemniscal recordings and for this reason they are considered as putative projecting neurons.

- Firstly, for each of the above seven profiles, neurons were grouped into groups or clusters and the neurons of the cluster closer to the LEM recordings were selected. For example, RRTF-LP recordings from PrV were grouped into three clusters; recordings in cluster 1 (denoted by a red line in Figure 5A, left) were the closest to LEM-LP ones. RRTF-BP recordings were also classified into three groups while TSR, MRL, and VS recordings in two. All selected clusters displayed a dynamic behavior very similar to that of lemniscal recordings in the whole 1–40 Hz stimulation range (Figure 5A, clusters in red).
- Secondly, we pooled together the neurons that were present to all selected clusters. This way we determined a subset of PrV recordings, each of which behaved similarly to the LEM recordings for all the above functions at all stimulation frequencies (15 out of 105, 14%, Figure 5B). Resulting recordings are tonic and thanks to the selection procedure probably correspond to whisker-related thalamic projecting PrV neurons, therefore rejecting the two channels hypothesis for the transmission of tactile information to the thalamus.

At this point we aimed at elucidating the origin of Ph and T behavior observed in PrV recordings.

### Origin of PrV phasic and tonic responses. cortical influence, neural modeling and simulations

Ph-T responses, and in particular T responses of trigeminothalamic projecting neurons, could be generated by two different types of PrV neurons (integrated of course in brainstem local circuits). However, we know that PrV neural dynamics are modulated by the somatosensory cortex (Sanchez-Jimenez et al., 2009). Consequently the tonic ad phasic behavior observed in our PrV recordings could be due (totally or partially) to the action of the corticotrigeminal projections on a single type of neuron. Removing of the cortical influence (by eliminating the sensorimotor cortex) let PrV neurons non-modulated.

To determine to which extent Ph-T responses are determined by the corticotrigeminal projecting neurons we applied our experimental protocol to 9 more animals after aspiration of the somatosensory cortex, obtaining 60 single-unit recordings. The removal of the somatosensory cortex leaded to an almost complete disappearance of Ph profiles (we recorded only 4 Ph cells and the Ph/T ratio passed from 0.37 in intact to 0.07 in decorticated animals).

**Figure 6:**
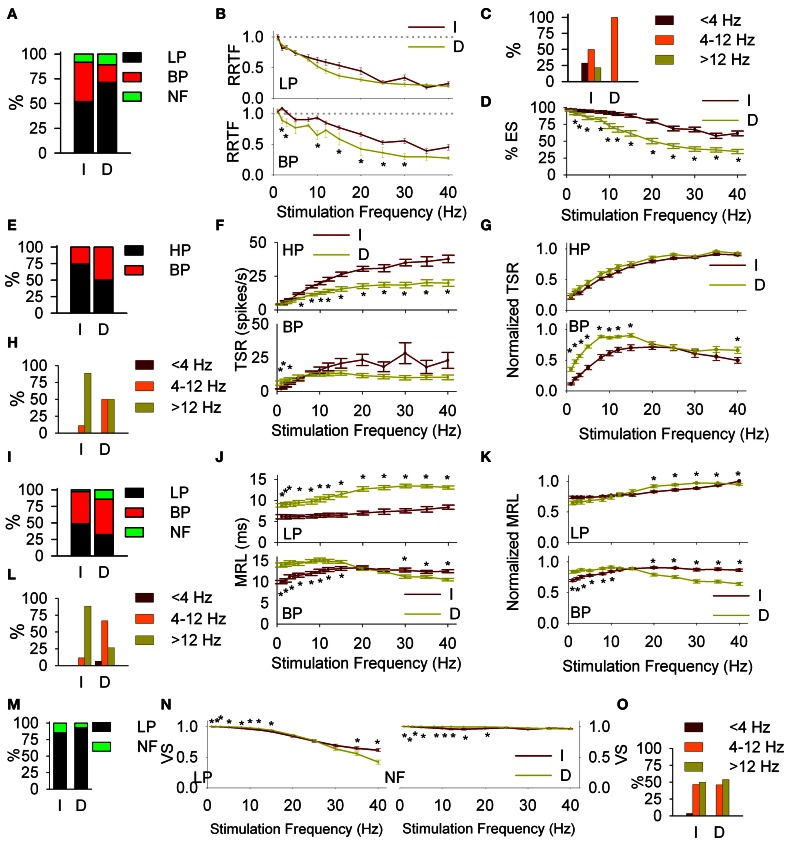
**Cortical modulation of PrV filtering properties**. Comparisons of intact (I) and decorticated (D) animals. Early response (RRTF): percentages of low-pass (LP) and band-pass (BP) responses **(A)**; mean values of LP and BP recordings **(B)**; distribution of BP frequencies in the three frequency ranges **(C)** and effectiveness of the stimulation **(D)**. Main differences are observed in the distribution of BP frequencies, BP mean values and effectiveness. Global response (TSR): percentages of HP and BP responses **(E)**; mean values of high-pass (HP) and BP recordings, crude **(F)** and normalized **(G)**; distribution of BP frequencies in the three frequency ranges **(H)**. Main differences are observed in BP mean values below whisking, LP into and above whisking and normalized BP up to 15 Hz. Latency (MRL): percentages of LP and BP responses **(I)**; mean values of LP and BP recordings, crude **(J)** and normalized **(K)**; distribution of BP frequencies in the three frequency ranges **(L)**. Differences are observed in almost all cases. Temporal consistency (VS): percentages of LP and NF responses **(M)**; mean values of LP and no-filtering (NF) recordings **(N)**; distribution of BP frequencies in the three frequency ranges **(O)**. Differences are mainly observed in mean VS values below and into whisking ranges. In all cases (^*^) indicate adjusted *p*-values < 0.05 (FDR procedure, 13 comparisons).

Significant changes were also induced in the dynamics of T cells for all RRTF, TSR, MRL and VS functions: **(1)** Early behavior: BP percentage decreased from 40 to 18%; BP-RRTF values decreased in the whole 1–40 Hz range; BP potentiation frequencies now fell exclusively into *W*_IN_ and effectiveness decreased for all stimulation frequencies (Figures 6A–D, some values are not significantly different due to the size of the sample). **(2)** Global behavior: BP percentage increased from 26 to 50%; HP-TSR values decreased in *W*_IN_−*W*_AB_ and normalized BP-TSR increased in *W*_BE_−*W*_IN_; BP potentiation frequencies are now equally distributed between *W*_IN_ and *W*_AB_ (Figures 6E–H). (3) Response latency: BP percentage increased from 49 to 54%; LP-MRL increased at all stimulation frequencies and BP-MRL below 20 Hz; BP potentiation frequencies shifted from *W*_AB_ to *W*_IN_ (Figures 6I–L). 4) Temporal consistency: VS increased for both, LP and NF recordings < 25 Hz (Figures 6M–O).

Disappearing of Ph profiles and alteration of T ones indicate the strong involvement of the cortex and suggest that Ph responses are generated by T neurons and modulated by the somatosensory cortex. However, data from decorticated animals alone do not clarify if trigeminal Ph and T profiles and T profiles of thalamic projecting neurons correspond to the same type or to two different types of neural cells. Consequently we tested the hypothesis that Ph and T profiles are generated by the same type of trigeminal neurons.

Our approach consisted in simulating the firing rates of these cells, perform extensive simulations and analyze the responses of the modeled neurons under the above experimental conditions.

**Figure 7:**
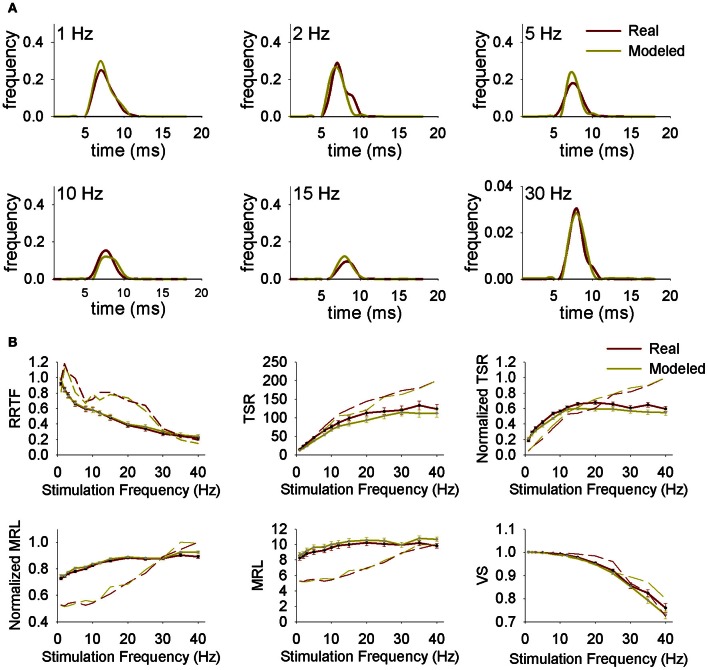
**(A)** Representative normalized peristimuli histograms at different stimulation frequencies from a real and a modeled neuron. **(B)** Repetition rate transfer function (RRTF), total spike response (TSR), mean response latency (MRL), and vector strength (VS) values at each stimulation frequency from real and modeled neurons. Continuous lines correspond to average values while dashed lines correspond to representative individual neurons.

First of all we simulated the spiking behavior of Ph and T trigeminal cells and thalamic projecting neurons using PrV and LEM experimental data from intact animals. Simulated PrV neurons accurately reproduced Ph-T responses at any stimulation frequency and RRTF, TSR, MRL, and VS values were similar to the real ones. Typical responses of real and simulated neurons and their RRTF, TSR, MRL, and VS values are shown in Figures 7A,B. The responses of the simulated neurons show significant correlations with those of real recordings in more than 90% of the cases. Significant *r*^2^ were always above 0.6 and in >70% of the cases above 0.9. Similar results were obtained in the simulations of projecting neurons and their comparison with the recordings from the medial lemniscus.

After that we simulated neural responses under decorticated conditions. Removal of the cortical input (excitatory, glutamatergic) could either increase or decrease the excitability of PrV neurons depending on the involved circuits and local inhibitory interneurons. In our simulations enhancement or reduction of neural excitability is achieved by increasing or decreasing *r*_0_(*t*) of each recording by the difference of mean *r*_0_(*t*) values at each bin between intact and decorticated animals.

**Figure 8:**
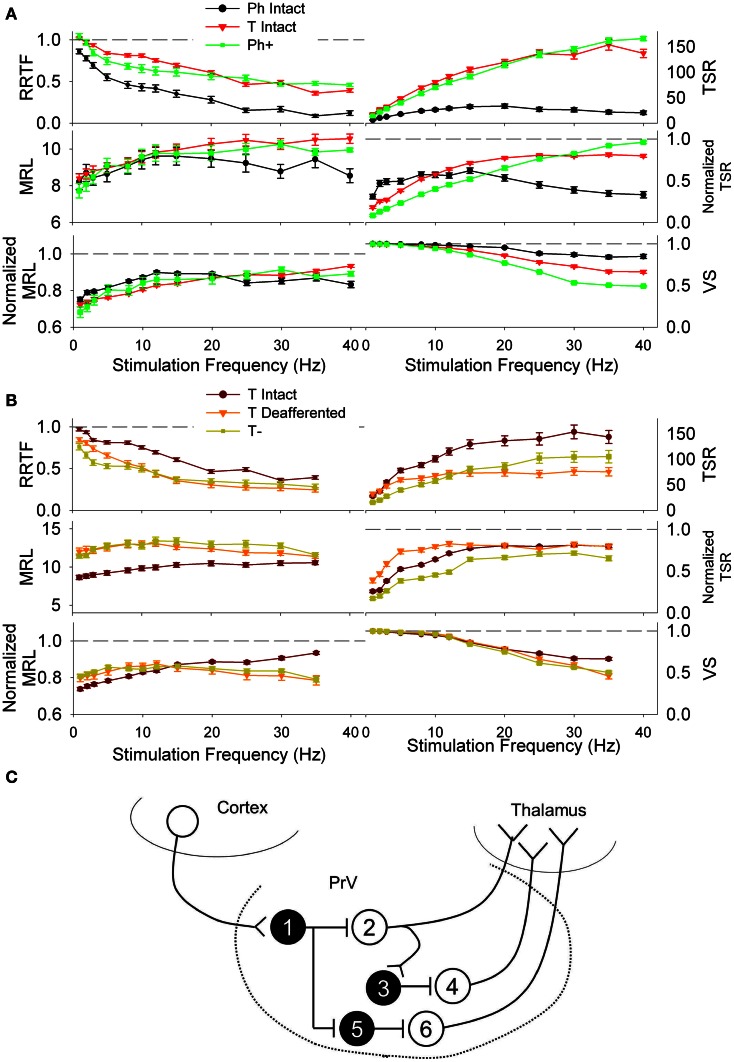
**(A)** Repetition rate transfer function (RRTF), total spike response (TSR), mean response latency (MRL), and vector strength (VS) values of phasic (Ph, black) and tonic PrV neurons (T, red) recorded from intact animals. Modeled deafferented phasic neurons (Ph+, green) behave similarly to the tonic ones when they are maintained in a high excitation state, suggesting they could be tonic neurons that fire phasically due to an inhibitory action of the corticotrigeminal projections (neuron 2 in the scheme below). **(B)** RRTF, TSR, MRL, and VS values of tonic intact (maroon) and tonic deafferented PrV neurons (T−, orange). Modeled deafferented tonic neurons (olive) behave similarly to the tonic intact ones when they are maintained in a low excitation state, suggesting they could be tonic neurons that fire phasically due to an excitatory action of the corticotrigeminal projections (neurons 4 and 6 in the scheme below). **(C)** Schematic representation of possible PrV intranuclear connections and cortical input determining tonic and phasic responses of the trigeminal neurons shown in **(A)** and **(B)**. Black filled, inhibitory interneurons (numbered 1, 3, and 5); white filled (neurons 2, 4, and 6), thalamic projecting tonic neurons responding phasically under the influence of the cortical input and tonically after decortication (neuron 2) or tonically under normal conditions and being inhibited after removal of the sensorimotor cortex (neurons 4 and 6).

High excitability Ph cells (denoted by Ph+) fitted well RRTF, TSR, VS, and MRL values of non-decorticated T cells (Figure 8A) while low excitability T neurons (denoted by T−) fitted well the values of decorticated T neurons at any of the stimulation frequencies (Figure 8B). Our results suggests that Ph responses recorded under normal conditions could be generated by T neurons maintained at a low excitability state by the corticotrigeminal input (e.g., neuron 2 in Figure 8C). Conversely, T responses could be generated by the same T neurons pushed at a high exitability state by the corticotrigeminal input (e.g., neurons 4 and 6 in Figure 8C). In the example of Figure 8C the removal of the corticotrigeminal input will enhance the excitability of neurons 2, 3, and 5 and reduce the excitability of 1, 4, and 6. Ph− and T+ neurons did not fit any of the experimental data.

**Figure 9:**
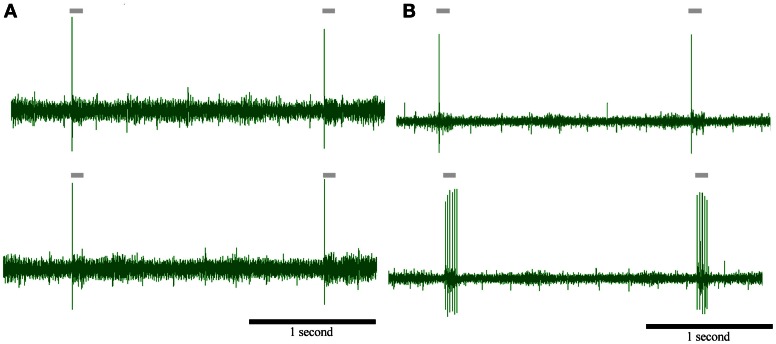
**Intense peripheral stimulation can transform a phasic neuron to tonic**. Top: responses of two phasic PrV neurons to peripheral stimulation of the principal whisker (control). Bottom: responses of the above phasic PrV neurons to peripheral stimulation of the principal whisker after intense stimulation of the same whisker (experimental). **(A)** neuron does not change its response while **(B)** neuron is transformed to tonic, probably, due to a depolarization of the membrane potential by the peripheral stimuli. Time scale (in seconds) is the same for all recordings.

As an ultimate proof that they are two responses by a single cell type we recorded neural responses of Ph cells after intense whisker stimulation. Intense peripheral stimulation could result in a depolarization of the membrane potential (otherwise hyperpolarized by the cortex) and transform Ph firing to T. We obtained 28 recordings of clearly phasic cells. Intense peripheral stimulation transformed phasic cells to tonic in 10 cases (Figure 9A) and did not alter the type of the response in 18 (Figure 9B).

## Discussion

Slowly adapting (T) and rapidly adapting (Ph) cells are present in all relay stations of the somatosensory system from peripheral receptors to the cortical pyramidal cells. In the peripheral nervous system T and Ph neurons process and transmit complementary information. However, no knowledge is available regarding the continuity of such segregation in the hierarchy of relay stations of the somatosensory pathway. In particular, although the two responses have been widely described in rat's PrV they have not been associated to some specific type of neurons (Shipley, 1974; Jacquin et al., 1993; Veinante and Deschenes, 1999; Sosnik et al., 2001; Minnery et al., 2003; Moreno et al., 2005).

Our results, both electrophysiological and numerical, advocate for complementary information processing by Ph and T trigeminal neurons; provide evidence that only tonic neural activity is used for tactile information coding and transmission to the thalamus; and show that Ph and T neurons do not correspond to two different neural cells but they arise from a single type of neurons under the influence of the somatosensory cortex.

Our data from individual PrV neurons are fully consistent with previous population data from our lab as both frequency-dependent potentiation/filtering and corticothalamic modulation of the trigeminal responses are concerned (Sanchez-Jimenez et al., 2009). PrV frequency-dependent processing of vibrissae-related sensory stimuli is also consistent with data from all relay stations of the lemniscal system (Sosnik et al., 2001; Castro-Alamancos, 2002a,b; Garabedian et al., 2003; Hartings et al., 2003; Moore, 2004; Sanchez-Jimenez et al., 2009).

### Complementary information processing by tonic and phasic neurons into the principal trigeminal nucleus

In early behavior Ph are mainly LP and T are either LP or BP but their mean values decay with the increasing of the stimulation frequency. Mean RRTF values of both BP types of neurons clearly potentiate below whisking frequencies and Ph ones filter in the whisking range although, individually, Ph-BP neurons potentiate in whisking and below whisking and T-BP in the whole 1–40 Hz range. The same filtering has been described in the ventral posterolateral nucleus of the thalamus (VPM) (Diamond et al., 1992; Fanselow and Nicolelis, 1999; Sosnik et al., 2001; Castro-Alamancos, 2002a; Deschenes et al., 2003; Hartings et al., 2003) and layer IV of the primary somatosensory cortex (SI) (Ahissar et al., 2000; Garabedian et al., 2003) indicating that early information processing is performed in a similar and fast way alongside the whole sensory pathway.

In global behavior Ph neurons are devoted to BP and T to HP filtering of incoming signals with T neurons performing only above whisking frequencies. This is consistent with our findings on tonic information transmission to the thalamus and experimental data from VPM neurons showing a high-pass filtering of the global response (Hartings et al., 2003) high pass filtering of SI barrel neurons reported in Lak et al. (2008) and Luna et al. (2005) although cortical band-pass filtering between 5–12 Hz has been reported by Garabedian et al. (2003).

In response latencies PrV neurons display the same behavior as in RRTF with T neurons being either LP or BP, mainly potentiating in the above whisking range. Although we observed T-BP potentiation transmitted to the thalamus, no relation between response latencies and stimulation frequency has been reported neither in VPM nor in SI (Sosnik et al., 2001; Garabedian et al., 2003; Moore, 2004).

In temporal consistency T neurons are almost exclusively LP or they don't perform potentiation/filtering while VPM and SI act as HP and BP filters respectively (Garabedian et al., 2003; Hartings et al., 2003; Moore, 2004) suggesting that spike timing is essential for information processing within the thalamocortical loop but this is not related to spiking precision into PrV.

Our results offer an answer to the question raised by Jones et al. (2004) who, despite the complementarity of slowly adapting and rapidly adapting primary sensory afferents, did not find differences in the timing and mean spiking rates of trigeminal Ph and T neurons and concluded that more sophisticated information processing mechanisms are needed for robust coding of time-varying tactile stimuli.

Decortication qualitatively modifies the type of the responses (T, Ph), filtering percentages (LP, BP, or HP) and BP filtering frequencies for each one of the four functions (RRTF, TSR, MRL, VS). However, cortical input is not responsible for the frequency-dependent behavior of PrV neurons since such decorticated PrV neurons still perform such processing although in a somewhat different way.

Our data from both decorticated animals and numerical simulations suggest that Ph and T responses do not correspond to two physiologically different cells but rather to two behavioral states of the same neurons. Cortical removal provokes both quantitative and qualitative changes in PrV neural responses. Quantitatively, decortication results in a decrease of excitability so PrV neurons show lower number of spikes, greater adaptation and longer latencies at most stimulation frequencies. Our findings are in agreement with Simons et al. (1992), Friedberg et al. (1999), Minnery and Simons (2003), and Moreno et al. (2005) on the decrease of PrV spontaneous activity and support the suggestion of Minnery and Simons (2003) that deeper levels of anesthesia mitigate the expression of PrV tonic responses. Decorticated single-unit responses in the present work are consistent with multi-unit responses observed under similar experimental conditions (Sanchez-Jimenez et al., 2009).

Transforming phasic responses to tonic provided additional support to the hypothesis that these neurons are tonic but are maintained hyperpolarized and modulated by the somatosensory cortex. The increased stimulation of the whiskers increased the electrical activity of the primary fibers which in turn probably increased the number and/or amplitude of the excitatory post synaptic potentials of the PrV phasic neuron. However, in most of the cases depolarization did not occur probably because peripheral stimulation also increased the inhibitory post synaptic potentials (directly or indirectly through feedback collaterals and/or via the spinal nuclei). In addition cortical influence could also be strong enough to maintain the membrane hyperpolarized.

### Information transmission to the thalamus

LEM electrophysiological data and in particular the dominance of tonic responses suggest information is transmitted by a unique trigeminothalamic channel. The statistically significant differences between LEM and PrV dynamics suggest trigeminothalamic responses correspond to a specific subset of T neurons. Cluster analysis corroborates this conclusion and the percentage of our putative projecting neurons is in agreement with the number of neurons for the barrelets region of the principal nucleus (Avendaño et al., 2005).

Tonic and phasic responses have also been observed in the lemniscal pathway (nuclei gracilis and cuneatus) (McComas, 1963) and complementary behavior has been described in type I (thalamic projecting) and type II (locally projecting) oscillating neurons (Panetsos et al., 1998). PrV barrelets region and lemniscal architectures are similar, based on large glutamatergic thalamic projecting neurons and smaller GABA-glycinergic interneurons (Valverde, 1966; Tan and Lieberman, 1978; Barbaresi et al., 1986; Avendaño et al., 2005). Our putative thalamocortical cells probably correspond to type I glutamatergic neurons.

These results do not strictly reject the two-channel-hypothesis, they do not support it and LEM recordings could be biased towards the tonic cell pathway. However, we can exclude this possibility because (1) trigeminothalamic projections are all traveling through the medial lemniscus (2) the diameter of the lemniscus in the coordinates of our recordings is very small and systematic sampling cannot miss one of the two putative responses (3) these results are in agreement with the literature [e.g., Chiaia et al. (2000) and Minnery and Simons (2003)] and (4) our simulations confirm cluster analysis results.

In general our lemniscal recordings are fully compatible with data from Chiaia et al. (2000) and Minnery and Simons (2003) that report up to 90% of T lemniscal fibers. In this context, information for both object location and object discrimination arriving from Ph and T Vg afferents is integrated by PrV neurons and transmitted to the thalamus by specific T neurons. This is in accordance with the tonic-to-phasic transformation that takes place in the thalamus (Hartings and Simons, 2000; Minnery and Simons, 2003).

The existence of a unique type of neuron that changes its state between T and Ph, that performs complementary information processing and coding depending on extranuclear or intranuclear variables and the transmission of tactile information to the thalamus coded into low-pass, band-pass and high-pass filtering profiles of exclusively T profiles could be involved in phenomena of neural plasticity and reorganization and even hyperalgesia and allodynia after peripheral deafferentation (Panetsos et al., 1995; Calford, 2002; Sandkuhler, 2009).

## Conclusions

In the present paper we provide evidence that phasic and tonic neurons in the first relay station of the rat trigeminal system (principal nucleus) process complementary aspects of whisker-related tactile information. Then we show that phasic and tonic firing patterns are not originated from two different types of neurons but they are due to the differential excitatory-inhibitory action of the sensorimotor cortex on a unique type of PrV cell. Finally we conclude that tonic and phasic responses do not constitute two different channels for the transmission of tactile information to the thalamus and that trigeminothalamic transmission is exclusively performed by tonically firing neurons. Our results could be important for both basic research on neural circuits and information processing, and for clinical/therapeutic applications and implants of sensory neuroprostheses.

### Conflict of interest statement

The authors declare that the research was conducted in the absence of any commercial or financial relationships that could be construed as a potential conflict of interest.
